# Complete plastome data of *Saccharum* hybrid cultivar VMC 76–16, a valuable genomic resource for investigating evolutionary relationships among modern sugarcane cultivars in Indonesia

**DOI:** 10.1016/j.dib.2026.113115

**Published:** 2026-07-30

**Authors:** Tiara Putria Judith, Widhi Dyah Sawitri, Oliv Nurul Kanaya, Boon Chin Tan, Kah Ooi Chua, Ganies Riza Aristya

**Affiliations:** a Department of Tropical Biology, Faculty of Biology, Universitas Gadjah Mada, 55281, Yogyakarta, Indonesia; b Department of Agronomy, Faculty of Agriculture, Universitas Gadjah Mada, 55281, Yogyakarta, Indonesia; c Centre for Research in Biotechnology for Agriculture (CEBAR), Universiti Malaya, 50603, Kuala Lumpur, Malaysia; d Institute of Biological Sciences (ISB), Faculty of Science, Universiti Malaya, 50603, Kuala Lumpur, Malaysia

**Keywords:** Dnbseq, Genomic, Phylogenetic, Plastome, Sugarcane

## Abstract

Sugarcane (*Saccharum* hybrid) is one of the world's most important sugar-producing crops and a valuable genomic resource for supporting molecular characterization and breeding programs. This article describes the complete chloroplast genome (plastome) dataset of the Indonesian sugarcane cultivar VMC 76–16, an introduced cultivar with desirable agronomic traits, including high sucrose content, early maturity, and tolerance to several major diseases. The plastome was assembled from paired-end DNBSEQ sequencing data comprising approximately 1.5 Gb of raw data generated from 5000,000 paired-end reads (2 × 150 bp). The assembled plastome is 141,181 bp in length with an overall GC content of 38.0% and exhibits the typical quadripartite structure, consisting of an 83,047 bp large single-copy (LSC) region, a 12,544 bp small single-copy (SSC) region, and two 22,795 bp inverted repeat (IR) regions. Genome annotation identified 135 genes, including 87 protein-coding genes, 40 transfer RNA genes, and eight ribosomal RNA genes. The dataset also includes comparative relative synonymous codon usage (RSCU) analysis of all available *Saccharum* plastomes retrieved from the NCBI database, simple sequence repeat (SSR) identification, and plastome-based phylogenetic analyses of *Saccharum* hybrid cultivar VMC 76–16 together with representative taxa of the tribe Andropogoneae. These data provide a genomic resource for comparative genomics, molecular characterization, phylogenetic analyses, plastome evolution studies, and future sugarcane breeding research.

Specifications Table

**Table:** 

Subject	Biology
Specific subject area	Omics: Genomics
Type of data	Table, Figure. Raw, Analyzed.
Data collection	Total genomic DNA from fresh leaf samples of *Saccharum* hybrid cultivar VMC 76–16 was extracted using a modified CTAB method. Library preparation was performed using the Hieff NGS® OnePot Pro DNA Library Prep Kit V4, followed by sequencing on the DNBSEQ-T7 platform. Raw reads were filtered using fastp v1.3.2. The complete plastome was assembled using GetOrganelle v1.7.7.1, annotated using GeSeq, and visualized using CPGView. Codon usage patterns were analyzed by calculating relative synonymous codon usage (RSCU) values using MEGA v11.0.13, and the resulting patterns were visualized using the RSCU-Plot Shiny application. Simple sequence repeats (SSRs) were identified using the web-based MISA tool, and phylogenetic analyses were conducted using IQ-TREE v3.1.1 and MrBayes v3.2.7, with trees visualized using iTOL v6.
Data source location	Location: PT Madubaru–PG Madukismo, Bantul Regency, Special Region of Yogyakarta Country: Indonesia Latitude and longitude: 7°49′40″S, 110°20′14″E
Data accessibility	Repository name: NCBI BioProject Data identification number: PRJNA1482419 Direct URL to data: https://www.ncbi.nlm.nih.gov/bioproject/PRJNA1482419 Repository name: NCBI BioSample Data identification number: SAMN61128509 Direct URL to data: https://www.ncbi.nlm.nih.gov/biosample/SAMN61128509 Repository name: NCBI Sequence Read Archive (SRA) Data identification number: SRR39385112 Direct URL to data: https://www.ncbi.nlm.nih.gov/nuccore/?term=SRR39385112 Repository name: NCBI GenBank Database Data identification number: PZ593130 Direct URL to data: https://www.ncbi.nlm.nih.gov/nuccore/PZ593130
Related research article	None.

## 1 Value of the Data

• The complete plastome sequence of *Saccharum* hybrid cultivar VMC 76–16, an introduced and officially released commercial sugarcane cultivar in Indonesia, provides a valuable genomic resource containing information on plastome organization, gene content, and structural characteristics. This dataset expands the available plastome resources of *Saccharum* hybrid cultivars and supports future comparative genomic and evolutionary studies.
• The identified simple sequence repeats (SSRs) provide potential molecular markers for cultivar discrimination, germplasm assessment, and marker development, while the relative synonymous codon usage (RSCU) profiles provide insights into codon preference patterns, plastome evolution, and plastid gene expression.
• This dataset provides a genomic reference for an Indonesian commercial sugarcane cultivar and can be utilized by molecular breeders, evolutionary biologists, and conservation geneticists for cultivar characterization, comparative analyses, and genetic resource assessment.
• The plastome-based phylogenetic framework generated from this dataset can further support studies on sugarcane diversity, evolutionary relationships, and genetic resource utilization for future breeding programs.

## 2 Background

Sugarcane (*Saccharum* hybrid) is an important industrial crop belonging to the genus *Saccharum* within the subtribe Saccharinae of the family Poaceae, which plays a major role in global sugar production [1]. The development of modern sugarcane cultivars has been largely influenced by conventional breeding programs that have contributed significantly to the improvement of agronomic traits since the Dutch colonial era in Indonesia [2]. Through selection, hybridization, and the introduction of elite germplasm, superior cultivars with desirable characteristics, such as high sucrose content, early maturity, disease resistance, and adaptability to diverse environments, have been developed [3]. One of the introduced cultivars is VMC 76–16 (*Victorias Milling Company*), originating from the Philippines and cultivated in several sugarcane-producing regions of Indonesia, including Java and Sulawesi. This cultivar exhibits valuable agronomic characteristics, including high sucrose content, early maturity, self-trashing ability, moderate ratooning capacity, and tolerance to several major sugarcane diseases such as pokkah boeng, mosaic, and leaf scald [4]. These characteristics highlight VMC 76–16 as an important genetic resource for supporting sugarcane improvement and breeding programs in Indonesia.

Among plant genomic resources, chloroplast genomes have become valuable datasets for species identification, comparative genomics, and molecular evolutionary studies [5]. The chloroplast genome, also known as the plastome, is typically composed of a circular double-stranded DNA molecule with a conserved quadripartite structure consisting of two inverted repeat (IR) regions and two single-copy regions, including the large single-copy (LSC) and small single-copy (SSC) regions [6,7]. In most plant species, plastomes are maternally inherited and exhibit relatively conserved genome organization, while still containing sufficient sequence variation for molecular characterization and evolutionary investigations [8]. The availability of complete plastome sequences enables the characterization of genome structure, gene content, conserved and variable regions, and potential molecular markers, such as simple sequence repeats (SSRs), which are valuable for genetic diversity assessment and phylogenetic reconstruction [9,10]. In sugarcane, plastome characterization provides a valuable genomic resource for understanding genetic relationships among *Saccharum* cultivars and supporting the evaluation of commercially important germplasm, including VMC 76–16, an introduced sugarcane cultivar widely cultivated in Indonesia.

## 3 Data Description

**Fig. 1:**
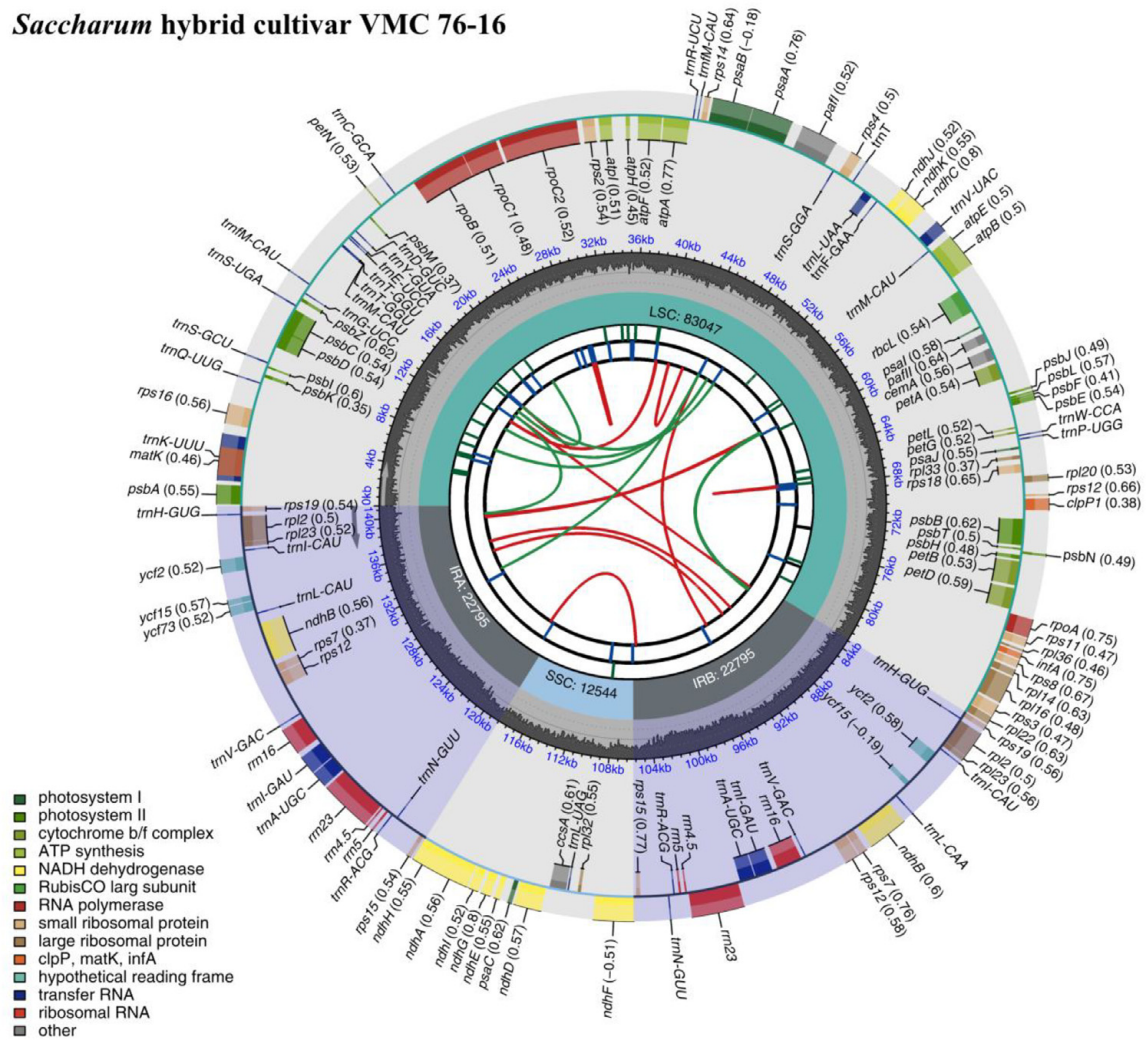
Plastome map of *Saccharum* hybrid cultivar VMC 76–16. Circular representation of the plastome showing gene organization, including protein-coding genes, tRNAs, rRNAs, and other genes. Genes located on the inner and outer sides of the circle are transcribed on the forward and reverse strands, respectively.

**Fig. 2:**
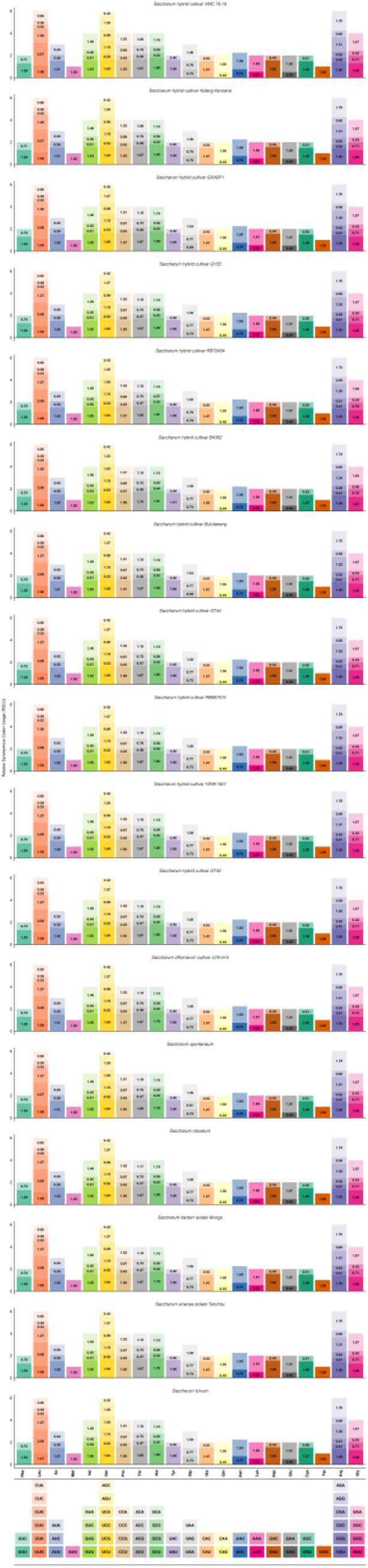
Comparative analysis of relative synonymous codon usage (RSCU) among all available *Saccharum* plastomes retrieved from the NCBI database. The taxa are arranged from top to bottom as follows: *Saccharum* hybrid cultivar VMC 76–16 (PZ593130), *Saccharum* hybrid cultivar Kidang Kencana (PZ344155), *Saccharum* hybrid cultivar GXASF1 (PV603803), *Saccharum* hybrid cultivar Q155 (NC_029221), *Saccharum* hybrid cultivar RB72454 (LN849914), *Saccharum* hybrid cultivar B4362 (BK010677), *Saccharum* hybrid cultivar Bululawang (PZ364438), *Saccharum* hybrid cultivar GT44 (PP454218), *Saccharum* hybrid cultivar RB867515 (KX507245), *Saccharum* hybrid cultivar YZ09–1601 (OR074484), *Saccharum* hybrid cultivar GT42 (PP454217), *Saccharum officinarum* cultivar IJ76–514 (NC_035224), *Saccharum spontaneum* (ON227480), *Saccharum robustum* (ON688645), *Saccharum barberi* isolate Mungo (NC_072524), *Saccharum sinense* isolate Tanzhou (OP896211), and *Saccharum fulvum* (OR268641).

**Fig. 3:**
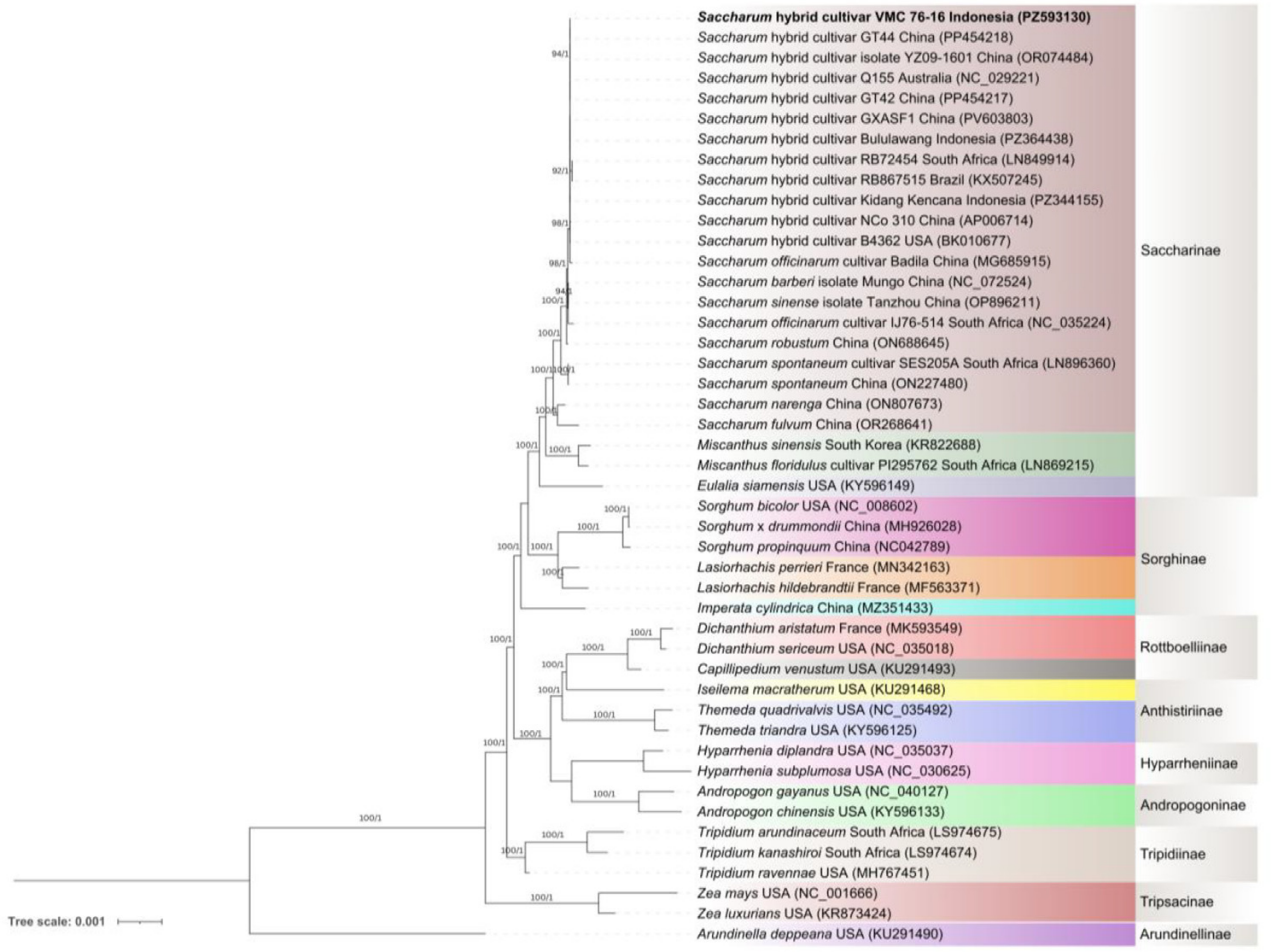
Phylogenetic tree reconstructed from complete plastome sequences using the Maximum Likelihood (ML) and Bayesian Inference (BI) methods, including *Saccharum* hybrid cultivar VMC 76–16, representative *Saccharum* species and hybrid cultivars, and selected members of the tribe Andropogoneae. Colored bars indicate the corresponding genera, while the gray boxes indicate their respective subtribes. *Arundinella deppeana* was used as the outgroup. Node labels represent ML bootstrap support values and BI posterior probabilities, respectively.

Genome sequencing generated approximately 1.5 Gb of raw paired-end data, consisting of 5000,000 paired-end reads (2 × 150 bp) with a GC content of 44.41%. After quality filtering, 9999,796 reads (99.99% of the total reads) were retained, with a mean read length of 149 bp, Q20 and Q30 values of 99.36% and 97.61%, respectively, and a GC content of 44.36%. The complete plastome of *Saccharum* hybrid cultivar VMC 76–16 was assembled into a circular DNA molecule of 141,181 bp, with an average sequencing coverage of 65.9 × and an overall GC content of 38.0%. The plastome exhibited the typical quadripartite structure, comprising a large single-copy (LSC) region of 83,047 bp, a small single-copy (SSC) region of 12,544 bp, and two inverted repeat (IRa and IRb) regions, each measuring 22,795 bp (Fig. 1). Genome annotation identified 135 genes, including 87 protein-coding genes, 40 transfer RNA (tRNA) genes, and eight ribosomal RNA (rRNA) genes (Fig. 1 and Table 1). The relative synonymous codon usage (RSCU) analysis showed a predominance of codons ending in adenine (A) or uracil (U) across all available *Saccharum* species and hybrid plastomes retrieved from the NCBI database (Fig. 2). Among the six synonymous codons encoding arginine, AGA consistently exhibited the highest RSCU value (1.74–1.76), whereas among the six synonymous codons encoding leucine, UUA exhibited the highest RSCU value (2.07–2.09), followed by CUU (1.25–1.28) across all analyzed *Saccharum* species and hybrid cultivars. A total of 32 mononucleotide simple sequence repeats (SSRs) were identified in the plastome of *Saccharum* hybrid cultivar VMC 76–16 (Table 2). Most SSRs were located in the LSC region (31, 96.88%), whereas only one SSR (3.12%) was detected in the SSC region. Based on genomic location, most mononucleotide SSRs were distributed in intergenic spacer (IGS) regions (24, 75.00%), followed by protein-coding genes (PCGs) (5, 15.63%) and introns (3, 9.38%). The identified SSR motifs were predominantly composed of A and T repeats, whereas only a single G mononucleotide SSR was detected. Repeat lengths ranged from 10 to 15 bp, with 10-bp A and T motifs being the most abundant. Phylogenetic analysis based on complete plastome sequences placed *Saccharum* hybrid cultivar VMC 76–16 in the same clade as *Saccharum* hybrid cultivar Kidang Kencana and *Saccharum* hybrid cultivar Bululawang from Indonesia, supported by 98% ML bootstrap and a Bayesian posterior probability of 1.00. The hybrid cultivars formed a distinct cluster within the *Saccharum* lineage together with *S. officinarum, S. spontaneum, S. robustum, S. barberi, S. sinense*, and *S. fulvum*, indicating a close evolutionary relationship among cultivated and wild *Saccharum* taxa. At a broader taxonomic level, the phylogeny clearly resolved the major subtribes of the tribe Andropogoneae, including Saccharinae, Sorghinae, Rottboelliinae, Anthistiriinae, Hyparrheniinae, Andropogoninae, Tripidiinae, and Tripsacinae, with *Arundinella deppeana* (Arundinellinae) serving as the outgroup (Fig. 3).

**Table 1:** Gene contents in the plastome of *Saccharum* hybrid cultivar VMC 76–16.

Functions	Family Genes	Name of Genes
Photosynthesis genes	Subunits of photosystem I	*psaA, psaB, psaC, psaI, psaJ*
	Subunits of photosystem II	*psbA, psbB, psbC, psbD, psbE, psbF, psbH, psbI, psbJ, psbK, psbL, psbM, psbN, psbT, psbZ*
	Subunits of cytochrome b6/f complex (electron transport chain)	*petA, petB*^1^*, petD*^1^*, petG, petL, petN*
	Subunits of ATP synthase	*atpA, atpB, atpE, atpF*^1^*, atpH, atpI*
	Subunits of Rubisco	*rbcL*
	Subunits of NADH-dehydrogenase (cyclic electron transport)	*ndhA*^1^*, ndhB*^1^^,*^, ndhC, ndhD, ndhE, ndhF, ndhG, ndhH, ndhI, ndhJ, ndhK
Self-replicating genes	Large subunit ribosomal proteins	*rpl2*^1^^,*^, rpl14, rpl16^1^, rpl20, rpl22, rpl23*, rpl32, rpl33, rpl36
	Small subunit ribosomal proteins	*rps2, rps3, rps4, rps7* *, rps8, rps11, rps12^#,*^, rps14, rps15 *, rps16^1^*, rps18, rps19* *
	DNA-dependent RNA polymerase	*rpoA, rpoB, rpoC1, rpoC2*
	Ribosomal RNA genes	*rrn23* *, rrn4.5 *, rrn5 *, rrn16 *
	Transfer RNA genes	*trnK-UUU*^1^*, trnQ-UUG, trnS-GCU, trnS-UGA, trnG-UCC, trnfM-CAU, trnT-GGU, trnM-CAU, trnE-UUC, trnY-GUA, trnD-GUC, trnC-GCA, trnR-UCU, trnS-GGA, trnT-UGU, trnL-UAA*^1^*, trnF-GAA, trnV-UAC*^1^, trnW-CCA, trnP-UGG, trnH-GUG*, trnI-CAU *, trnL-CAA *, trnV-GAC *, trnI-GAU^1^^,*^, trnA-UGC^1^^,*^, trnR-ACG*, trnN-GUU *, trnL-UAG
Other genes	Envelope membrane protein	*cemA*
	Translational initiation factor	*infA*
	c-type cytochrome synthesis gene	*ccsA*
	ATP-dependent protease (proteolytic function)	*clpP1*
	Maturase	*matK*
Genes of unknown functions	Conserved hypothetical chloroplast reading frames	ycf2 *, ycf15 *, ycf73 *

Notes: *Two gene copies in IRs; # Trans-splicing gene.

1 Gene containing one intron.

**Table 2:** Distribution of simple sequence repeats (SSRs) in the plastome of *Saccharum* hybrid cultivar VMC 76–16.

SSR Type	Motif	Repeat Number	Repeat Length (bp)	SSR Count	Region	Location
Mono-nucleotide	A	10	10	6	LSC	IGS (4)
						PCG (1, *rpoC2*)
					SSC	IGS (1)
	A	11	11	1	LSC	IGS (1)
	A	12	12	1	LSC	IGS (1)
	A	15	15	1	LSC	Intron (1, *matK-trnK-UUU*)
	T	10	10	10	LSC	IGS (7)
						Intron (1, *atpF*_1*-atpF*_2)
						PCG (2, *infA*)
	T	11	11	6	LSC	IGS (6)
	T	12	12	3	LSC	IGS (2)
						Intron (1, *rpl16*_2-*rpl16*_1)
	T	14	14	3	LSC	IGS (2)
						PCG (1, *ndhK*)
	G	10	10	1	LSC	PCG (1, *psbC*)
	Total			32	LSC (31, 96.88%) SSC (1, 3.12%)	IGS (24, 75%) Intron (3, 9.38%) PCG (5, 15.63%)

Notes: PCG, protein-coding genes; IGS, intergenic spacer; LSC, large single-copy region; and SSC, small single-copy region.

## 4 Experimental Design, Materials and Methods

### 4.1 Plant collection and DNA extraction

Plant samples of the *Saccharum* hybrid cultivar VMC 76–16, an officially released modern commercial sugarcane cultivar in Indonesia (Indonesian Ministry of Agriculture Decree No 3676/Kpts/SR.120/10/2010), were collected from PT Madubaru-PG Madukismo collection field, Bantul Regency, Special Region of Yogyakarta, Indonesia (7°49′40″S, 110°20′14″E). Fresh young leaves were immediately frozen in liquid nitrogen and stored at −20 °C until DNA extraction to preserve DNA integrity. Total genomic DNA was isolated using a modified cetyltrimethylammonium bromide (CTAB) method. DNA integrity was assessed by 0.7% agarose gel electrophoresis, showing a distinct high-molecular-weight DNA band with minimal smearing. DNA purity was evaluated using a NanoDrop One UV–Vis spectrophotometer (Thermo Fisher Scientific, USA), yielding a DNA concentration of 328.08 ng/µL with an A260/A280 ratio of 1.72. Accurate DNA quantification was further performed using a Qubit 3.0 Fluorometer (Thermo Fisher Scientific, USA), resulting in a concentration of 56.40 ng/µL.

### 4.2 Genome sequencing

DNA libraries were prepared using the Hieff NGS® OnePot Pro DNA Library Prep Kit V4 (12,972). Genomic DNA was fragmented and size-selected to enrich DNA fragments of approximately 200–400 bp, followed by end repair, A-tailing, adapter ligation, PCR amplification, purification, and circularization to generate single-stranded circular DNA libraries. The prepared libraries were quantified prior to sequencing on the DNBSEQ-T7 platform (MGI Tech Co., Ltd., China). Circular DNA libraries were amplified during sequencing through rolling circle replication to generate DNA nanoballs (DNBs), which were loaded onto high-density nanochips and sequenced using combinatorial Probe-Anchor Synthesis (cPAS) technology to generate paired-end sequencing reads.

### 4.3 Plastome assembly and annotation

Raw sequencing reads were processed using fastp v1.3.2 with default parameters for quality control, adapter trimming, and removal of low-quality reads [11]. The filtered reads were used for plastome assembly using GetOrganelle v1.7.7.1 [12]. The assembled plastome was evaluated using Bandage v0.8.1 to confirm the circular contig structure and the characteristic quadripartite organization of the plastome [13]. The assembled plastome was further validated through BLASTn analysis (https://blast.ncbi.nlm.nih.gov/Blast.cgi) against the NCBI database. Genome annotation was performed using GeSeq with manual curation. The annotated plastome was visualized using CPGView to generate the circular genome map and evaluate gene organization [14].

### 4.4 Codon usage analysis and simple sequence repeats detection

Codon usage patterns within the plastome were analyzed based on relative synonymous codon usage (RSCU) values. RSCU values were calculated using MEGA v11.0.13 [15], and the resulting codon usage profiles were visualized using the RSCU-Plot Shiny application (https://pcg-lab.shinyapps.io/RSCU-Plot/). Simple sequence repeats (SSRs) within the plastome sequence were identified using the web-based MISA tool (https://webblast.ipk-gatersleben.de/misa/).

### 4.5 Phylogenetic analysis

The newly assembled plastome sequence of *Saccharum* hybrid cultivar VMC 76–16 together with related plastome sequences retrieved from the NCBI GenBank database were used for phylogenetic analysis. Multiple sequence alignment of complete plastome sequences was performed using MAFFT v7.525 with automatic FFT-NS-2 strategy selection [16]. Ambiguously aligned regions and excessive gaps were removed using trimAl v1.5.1 [17]. Phylogenetic reconstruction was performed using Maximum Likelihood (ML) and Bayesian Inference (BI) approaches. Maximum Likelihood analysis was conducted using IQ-TREE v3.1.1 with automatic model selection implemented in ModelFinder and 1000 ultrafast bootstrap replicates [18,19]. The best-fit nucleotide substitution model was selected based on the Bayesian Information Criterion (BIC). Bayesian Inference analysis was performed using MrBayes v3.2.7 under the GTR+I+G substitution model with 10,000,000 MCMC generations, two independent runs, and four Markov chains [20]. Trees and parameters were sampled every 100 generations, with the first 25% of sampled trees discarded as burn-in. The resulting ML and BI phylogenetic trees were visualized using iTOL v6 (https://itol.embl.de).

## Limitations

‘None’.

## Ethics Statement

All authors have read and followed the ethical requirements for publication in Data in Brief and confirm that the current work does not involve human subjects, animal experiments, or any data collected from social media platforms.

## Credit Author Statement

**Tiara Putria Judith:** Investigation, Methodology, Formal Analysis, Data Curation, Visualization, Writing – Original Draft. **Widhi Dyah Sawitri:** Supervision, Funding acquisition, Writing – Review & Editing. **Oliv Nurul Kanaya:** Resources, Investigation. **Boon Chin Tan:** Methodology, Validation, Software. **Kah Ooi Chua:** Methodology, Validation, Software. **Ganies Riza Aristya:** Supervision, Project Administration, Funding acquisition, Conceptualization, Writing – Review & Editing.

## Data Availability

(NCBI).Saccharum hybrid cultivar VMC 76-16 chloroplast, complete genome (Original data)

## Appendix Supplementary materials

Image, application 1
